# Effects of Jianpi Yiqi method for chronic obstructive pulmonary disease: A protocol for systematic review and meta-analysis

**DOI:** 10.1097/MD.0000000000020566

**Published:** 2020-06-12

**Authors:** Yuxi Wang, Yihui Jiang, Junchen Ge, Zhiwei Zhang, Jia Dong

**Affiliations:** a Hangzhou Lin’ an TCM Hospital; b People's Liberation Army No. 962 Hospital, China.

**Keywords:** chronic obstructive pulmonary disease, Jianpi Yiqi method, protocol, systematic review and meta-analysis

## Abstract

**Background:**

Chronic obstructive pulmonary disease (COPD) is a chronic inflammatory lung disease with high morbidity and mortality worldwide. Jianpi Yiqi method (JYM) is a classical Chinese therapy in the treatment of COPD. However, there is no systematic review related to JYM for COPD. In this study, we aim to systematically examine the efficacy and safety for clinical use of JYM.

**Methods and analysis:**

We will conduct a comprehensive retrieval in the following electronic databases: PubMed, Embase, MEDLINE, Cochrane Library Central Register of Controlled Trials, China National Knowledge Infrastructure (CNKI) database, Wanfang Data Knowledge Service Platform, Chinese Scientific Journals Database (VIP), Chinese Biomedical Literature Service System (SinoMed), and other sources. Two trained reviewers will identify relevant studies, extract data information, and then assess the methodical quality by the Cochrane risk of bias assessment tool, independently. Then the meta-analyses will be conducted by using the RevMan 5.2. Based on the heterogeneity test, data integration is performed using a fixed effect model or a random effects model. A sensitivity analysis will be performed to evaluate the stability of the results. Then publication bias assessment will be conducted by funnel plot analysis and Egger test. Finally, the quality of evidence will be assessed by the GRADE system.

**Results:**

The results of our research will be published in a peer-reviewed journal.

**Conclusion:**

The conclusion of our systematic review will provide evidence to judge whether JYM is an effective intervention for patients with COPD.

**OSF registration number:**

10.17605/OSF.IO/JKQYV.

## 1 Introduction

Chronic obstructive pulmonary disease (COPD) is a chronic inflammatory lung disease that causes obstructed airflow from the lungs. It is a serious public health problem that ranks as the fourth leading cause of death around the word.^[1]^ Based on a national cross-sectional study in China, the overall incidence of spirometry-defined COPD was 8.6%, accounting for about 99.9 million people.^[2]^ Therefore, effective treatment of COPD is of great significance to increase patients quality of life and decrease the social and economic burden. Currently, bronchodilators, glucocorticoids, and theophylline are regarded as the main drugs for the treatment of COPD.^[3]^ These therapies have been proved to be efficient in relieving symptoms, reducing the frequency, and severity of exacerbations.^[4,5]^ However, many patients still suffered from dyspnea and substantial limitations in daily life. Moreover, these therapies have been associated with some side effects such as hoarseness, candidiasis, and risk of pneumonia,^[6,7]^ which greatly restricts the treatment of COPD. Therefore, seeking complementary and alternative treatments for COPD is highly significant.

Traditional Chinese medicine (TCM) has been used to treat COPD for its multiple targets and systematic regulation. Chinese patients with COPD usually take TCM to treat the disease owing to its efficacy, safety, low cost, and sustainable improvement of patients symptoms and prognosis. It has been proven that the use of TCM is effective in improving clinical effective rates, lung function, and quality of life.^[8]^ Based on the characteristics of TCM-defined syndromes of COPD, qi deficiency was considered to be the key pathogenetic factor.^[9]^ Thus, Jianpi Yiqi method (JYM) should be incorporated into the treatment of COPD. Clinical and experimental studies have confirmed that JYM can reduce the frequency of acute exacerbations, improve the quality of life, and reduce the severity of inflammation.^[9–11]^ However, there is still a lack of systematic review to analyze the clinical evidence of JYM in the treatment of COPD. Therefore, a comprehensive review is urgently needed to support the effectiveness and safety of JYM on patients with COPD. In this work, we will summarize the existing evidence and evaluate the efficacy and safety of JYM for COPD.

## 2 Methods and analysis

Our work has been registered at Open Science Framework (OSF, https://osf.io/). The registration DOI of this study is 10.17605/OSF.IO/JKQYV. This protocol will be conducted followed the guideline of the Preferred Reporting Items for Systematic Review and Meta-Analysis Protocols (PRISMA-P) recommendations.^[12]^

### 2.1 Eligibility criteria

#### 2.1.1 Types of studies

In this work, randomized controlled trials (RCTs) that used JYM or a combination of JYM and routine pharmacotherapy as treatment measures will be eligible. Non-randomized control studies and observational study will be excluded in the review. The language will be limited to English and Chinese.

#### 2.1.2 Type of participants

This study will include patients with COPD as per the criteria for diagnosis based on the Global Initiative for Chronic Obstructive Lung Disease (GOLD),^[13]^ or guideline of Chinese Medical Association Respiratory Diseases Society for COPD.^[14]^ The study will include all types of patients regardless of age, gender, region, and other factors. Patients who suffered from other respiratory system diseases will be excluded.

#### 2.1.3 Types of interventions and comparisons

We will only include studies which interventions involved JYM alone or combined with conventional therapy, as well as those with control groups that can verify the therapeutic effect of JYM. Studies that included other TCM therapies such as acupuncture, moxibustion, acupoint application with herbal medicine, and so forth will be excluded. There will be no limitation on the duration, specific composition, and dosage of JYM.

### 2.2 Types of outcome measures

#### 2.2.1 Primary outcomes

1. Frequency and duration of acute exacerbation of COPD during follow-up after study entry.
2. Lung function, including the FEV1, FVC, FEV1/predicted value (FEV1%), and FEV1/FVC.

#### 2.2.2 Secondary outcomes

1. Quality of life assessed by St George's Respiratory Questionnaire (SGRQ).
2. Clinical effective rates.^[8]^
3. Scores of TCM syndrome.^[8]^

### 2.3 Search strategy

#### 2.3.1 Electronics searches

To identify all relevant studies, RCTs will be searched primarily in PubMed, Embase, MEDLINE, Cochrane Library Central Register of Controlled Trials, China National Knowledge Infrastructure (CNKI) database, Wanfang Data Knowledge Service Platform, Chinese Scientific Journals Database (VIP), Chinese Biomedical Literature Service System (SinoMed) from their respective inception to April 2020. Two authors (Yuxi Wang and Yihui Jiang) will search and screen all the citations independently. The equivalent search words will be used in the Chinese databases. The search strategy for PubMed is as follows:

1#: Search (((((((((Lung Diseases, Obstructive[MeSH Terms]) AND Pulmonary Disease, Chronic Obstructive[MeSH Terms]) AND chronic obstructive pulmonary disease[Title/Abstract]) AND COPD[Title/Abstract]) AND chronic obstructive lung disease[Title/Abstract]) AND chronic obstructive airway disease[Title/Abstract]) AND chronic obstructive respiratory disease[Title/Abstract]) AND chronic airflow limitation[Title/Abstract]) AND chronic obstructive airway disease[Title/Abstract]) AND airflow obstruction[Title/Abstract]

2#: Search ((((((((((((Medicine, Chinese Traditional[MeSH Terms]) AND Herbal Medicine[MeSH Terms]) AND Drugs, Chinese Herbal[MeSH Terms]) AND traditional Chinese medicine[Title/Abstract]) AND Chinese medicine[Title/Abstract]) AND Chinese herb[Title/Abstract]) AND jianpi yiqi[Title/Abstract]) AND jianpi[Title/Abstract]) AND yiqi[Title/Abstract]) AND strengthen the spleen[Title/Abstract]) AND replenish qi[Title/Abstract]) AND invigorate spleen [Title/Abstract]) AND reinforce qi [Title/Abstract].

3#: Search ((((((((((randomized controlled trial[Title/Abstract]) OR RCT[Title/Abstract]) OR random[Title/Abstract]) OR randomly[Title/Abstract]) OR random allocation[Title/Abstract]) OR allocation[Title/Abstract]) OR randomized control trial[Title/Abstract]) OR controlled clinical trial[Title/Abstract]) OR clinical trial[Title/Abstract]) OR clinical study[Title/Abstract]

#1 and #2 and #3

#### 2.3.2 Searching other resources

Studies from other sources will also be obtained from the following sources:

1. Google scholar and Baidu scholar.
2. Chinese Clinical Trial Registry (ChiCTR).
3. ClinicalTrials.gov.

### 2.4 Selection of studies

**Figure 1:**
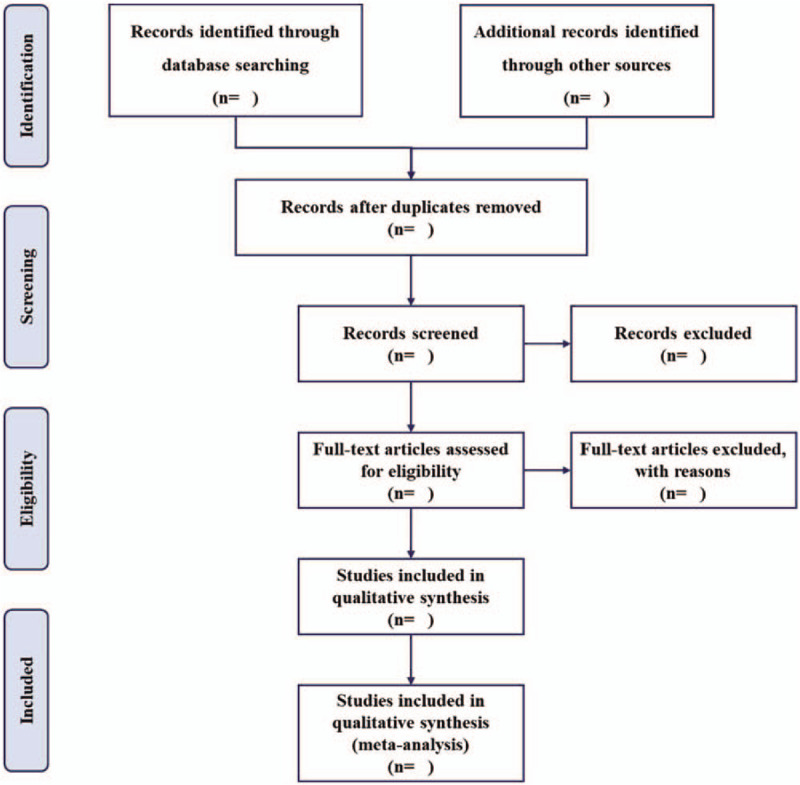
Flow chart of study selection.

The electronic citations extracted out from the above databases will be integrated into EndNote X9.0 software. Two methodological trained reviewers (Yuxi Wang and Yihui Jiang) will independently access the titles and abstracts of all searched studies in accordance with the established selection criteria. If necessary, the full-text papers will be downloaded and reviewed. Disagreements will be resolved through discussion with other authors. The excluded studies will be shown in a table with reasons. A PRISMA flow chart will be drawn to illustrate the study selection procedure (Fig. 1).

### 2.5 Data extraction and management

Two review authors (Yuxi Wang and Yihui Jiang) will independently check the information extracted from all eligible studies, and then import data into Excel tables. The following items will be extracted from individual studies: publication year, sample size, TCM therapeutic principle, details of treatment and control interventions, time of treatment, frequency and duration of acute exacerbation, lung function, clinical effective rates, TCM syndrome, quality of life, and adverse events. If data are not available from the studies, the missing information will be obtained by contacting the corresponding authors.

### 2.6 Assessment of risk of bias

The methodological quality of the RCTs will be evaluated by 2 independent reviewers (Yuxi Wang and Yihui Jiang) using the Cochrane Collaborations tool.^[15]^ The risk of bias of a trial is assessed through 6 items, including selection bias, performance bias, detection bias, attrition bias, reporting bias, and other sources of bias. These items will be classified into 3 levels: “Low risk”, “High risk” or “Unclear risk”. Disagreements will be discussed and arbitrated by all reviewers.

### 2.7 Measures of treatment effect

RevMan 5.2 (Cochrane, London, UK) provided by the Cochrane Collaboration will be applied for data analysis. For dichotomous variables, a risk ratio (RR) with 95% confidence interval (CI) will be used for analysis. For continuous variables, a mean difference (MD) or a standard mean difference (SMD) with 95% CIs will be used for analysis.

### 2.8 Assessment of heterogeneity

The Cochrane *X*^*2*^ and *I*^*2*^ tests will be applied to assess the statistical heterogeneity of evidence.^[16]^ The fixed effect model will be used to estimate the effect amount when *P* ≥ .05 and *I*^2^ ≤ 50%. The random effect model will be used when *P* < .05 and *I*^*2*^ > 50%.

### 2.9 Sensitivity analysis

Sensitivity analysis is usually used to analyze the quality of research, methodological elements, type of publication, etc. In this work, a sensitivity analysis will be performed to access the robustness of the results. We will exclude each study that is included in the analysis one by one, and then re-analyze and compile the data. The difference between the re-obtained effects and the original effects will be compared.

### 2.10 Publication bias assessment

When more than 10 studies are included, a funnel plot will be used to detect report bias. Egger test will be conducted for statistical investigation.^[17]^

### 2.11 Grading the quality of evidence

We will use the Grading of Recommendations Assessment, Development, and Evaluation (GRADE) to evaluate the quality level of evidence.^[18]^ The GRADE assessment classifies the quality of evidence into 4 levels: high, moderate, low, and very low.

### 2.12 Patient and public involvement

Patient and public were not involved in this study.

### 2.13 Ethics and dissemination

Ethical approval will not be required for this systematic review. The results of this review will be disseminated by being published in a peer-reviewed journal.

## 3 Discussion

COPD has been a major public problem, which is associated with high mortality rates and social burdens. Therefore, enough attention should be paid in the management of COPD. Studies have shown that Chinese medicine has beneficial effects on symptoms and quality of life of COPD patients.^[19]^ JYM is a classical Chinese therapy in treating COPD.^[20]^ However, the current state of evidence of JYM for COPD has been unknown. This will be the first review to compare the effectiveness of JYM for the treatment of COPD. We hope that the results of our study will promote evidence-based for clinical Chinese medicine.

## 4 Amendments

If amendments are needed, we will update our protocol to include any changes in the whole process of research.

## Author contributions

**Data curation:** Yuxi Wang, Yihui Jiang.

**Formal analysis:** Junchen Ge, Zhiwei Zhang, Jia Dong.

**Methodology:** Yuxi Wang, Yihui Jiang.

**Project administration:** Yuxi Wang.

**Software:** Yuxi Wang, Yihui Jiang

**Supervision:** Zhiwei Zhang, Jia Dong.

**Validation:** Junchen Ge.

**Visualization:** Junchen Ge.

**Writing – original draft:** Yuxi Wang, Yihui Jiang.

**Writing – review & editing:** Yuxi Wang, Junchen Ge.
